# Thrice-weekly post-haemodialysis ceftazidime can achieve adequate pre-dialysis concentrations and clinical cure in patients with melioidosis

**DOI:** 10.1093/jac/dkaf383

**Published:** 2025-10-13

**Authors:** Simon Smith, Christopher W Reilly, James Stewart, Thomas Ragh, Vanessa J Muller, Ibrahim Ismail, Josh Hanson

**Affiliations:** Department of Medicine, Cairns Hospital, Cairns, Queensland, Australia; Department of Pharmacy, Cairns Hospital, Cairns, Queensland, Australia; Department of Medicine, Cairns Hospital, Cairns, Queensland, Australia; Department of Pharmacy, Cairns Hospital, Cairns, Queensland, Australia; Department of Pharmacy, Cairns Hospital, Cairns, Queensland, Australia; Department of Medicine, Cairns Hospital, Cairns, Queensland, Australia; Department of Medicine, Cairns Hospital, Cairns, Queensland, Australia; Kirby Institute, University of New South Wales, Sydney, New South Wales, Australia

## Abstract

**Background:**

Melioidosis is an opportunistic infection caused by *Burkholderia pseudomallei*. Prolonged IV antibiotic therapy is required to cure the disease. Current guidelines recommend daily ceftazidime for people receiving chronic intermittent haemodialysis (CIHD).

**Methods:**

We reviewed all cases of culture-confirmed *B. pseudomallei* infection from 2016 to 2025. In patients receiving CIHD, we recorded their weekly CIHD schedule, their ceftazidime dosing regimen, the ceftazidime MIC for their initial *B. pseudomallei* isolate and all pre-dialysis ceftazidime concentrations.

**Results:**

There were 449 cases of melioidosis; 26 (5.8%) occurred in people already receiving—or who were commenced on—CIHD during their hospital admission. Of these, 24 (92%) survived to the completion of their intensive IV phase of antibiotic therapy. There were nine who had ceftazidime pre-dialysis concentrations measured. Of these, eight were commenced on ceftazidime 2 g daily, but five had their dosing regimen changed; this was a daily dose reduction in three and a change to thrice-weekly dosing regimen of 2 g/2 g/3 g post-haemodialysis in two. One patient was administered thrice-weekly dosing post-haemodialysis for the duration of their intensive phase. No cases had evidence of neurotoxicity, all received oral eradication therapy, and all had clinical cure.

**Conclusions:**

In patients receiving thrice-weekly CIHD, post-haemodialysis ceftazidime administered 2 g/2 g/3 g achieved adequate serum pre-dialysis concentrations, can achieve good clinical outcomes and may be a potential treatment approach for people with melioidosis. The availability of therapeutic drug monitoring and clinical breakpoints suggest a more individualized approach is possible in people with melioidosis receiving CIHD.

## Introduction

Melioidosis is a tropical, potentially life-threatening opportunistic infection caused by the environmental, Gram-negative bacterium *Burkholderia pseudomallei*. People with diabetes mellitus, hazardous alcohol use, chronic lung disease, chronic kidney disease, malignancy or immunosuppression are most at risk for the disease.^1^ Treatment of melioidosis is complex and consists of two phases: an intensive phase of either IV meropenem or ceftazidime, followed by an eradication phase, with oral trimethoprim/sulfamethoxazole being the agent of choice.^2^

Challenges with adherence to—and tolerance of—long-term high-dose oral trimethoprim/sulfamethoxazole have led to an increasingly prolonged intensive phase. It is recommended that all patients now receive between 2 and 8 weeks of IV therapy, typically administered through a peripherally inserted central catheter (PICC).^3^ IV meropenem is usually reserved for patients with melioidosis admitted to the ICU or for those with neuromelioidosis.^3^ For all other patients, IV ceftazidime is recommended, including those managed with outpatient parenteral antimicrobial therapy (OPAT) via elastomeric infusers.^4^

The optimal bactericidal activity of ceftazidime and other cephalosporins occurs at trough concentrations greater than four times the MIC.^5^ In Australia, most initial *B. pseudomallei* isolates have a ceftazidime MIC of <4 mg/L^6^ and current melioidosis treatment guidelines recommend weight-based dosing of ceftazidime at a dose of 50 mg/kg, up to 2 g 6-hourly with dose adjustment by calculated creatine clearance (CL_CR_).^7^ Patients receiving chronic intermittent haemodialysis (CIHD) are recommended to receive the same dose as those with CL_CR_ of <15 mL/min—2 g 24-hourly administered to people weighing >60 kg and 1 g 24-hourly to those weighing <60 kg.^7^

Ceftazidime is dependent upon glomerular filtration for elimination.^8^ The effective pharmacodynamic activity of ceftazidime occurs at free (unbound) drug concentrations exceeding the MIC for >45% of the interdialytic interval (*fT*_>MIC_) with maximal effects requiring 60%–70% *fT*_>MIC_.^9,10^ In people receiving thrice-weekly CIHD, this can be achieved by administering a dose of 2 g post-haemodialysis for a 48 h interdialytic period and 3 g post haemodialysis for a 72 h interdialytic period (2 g/2 g/3 g), although there are limited clinical data to support this approach.^11,12^

In patients with melioidosis receiving CIHD, thrice-weekly post-dialysis ceftazidime is an attractive option, particularly as it negates the need for PICC insertion and for daily OPAT visits. This study examined ceftazidime pre-dialysis concentrations and the clinical course of patients treated for melioidosis who were receiving CIHD.

## Methods

We performed this study at Cairns Hospital in Far North Queensland, tropical Australia. We reviewed all cases of culture-confirmed *B. pseudomallei* infection managed at the hospital from 1 January 2016 to 31 March 2025 and recorded data as described previously.^13^ These dates were chosen as they coincided with the establishment of an ongoing prospective observational study. In patients receiving CIHD, we recorded the ceftazidime MIC—defined by ETEST—of their initial *B. pseudomallei* isolate (EUCAST), their weekly CIHD schedule and their ceftazidime dosing regimen. We also recorded all pre-dialysis free (unbound) ceftazidime concentrations—if they had been performed—in these patients. The target range for unbound ceftazidime concentrations was defined as ≥1 × MIC. Ceftazidime concentrations for therapeutic drug monitoring became more readily available in 2018, with testing occurring at a reference laboratory in Brisbane, 1600 km from Cairns. The study was approved by the Far North Queensland Human Research Ethics Committee (HREC/15/QCH/46-977). As the retrospective data were de-identified, the Committee waived the requirement for informed consent.

## Results

There was a total of 449 cases of melioidosis during the study period; 21 (4.6%) occurred in people already receiving CIHD and 5 (1.1%) occurred in people with preexisting chronic kidney disease who initiated CIHD during their illness. Of these 26 cases, 2 (8%) died from multi-organ failure within 3 days of their initial diagnosis of melioidosis despite admission to ICU. The remaining 24 (92%) survived to completion of their intensive phase of antibiotic therapy, 9 of whom had at least one ceftazidime pre-dialysis concentration measured (Table 1). Of these nine cases, eight were initially commenced on ceftazidime 2 g daily, but five had their dosing regimen changed. This was a daily dose reduction in three and a change to thrice-weekly dosing regimen of 2 g/2 g/3 g administered immediately post-haemodialysis in two, one of whom had ceftazidime pre-dialysis concentrations performed. One patient was administered thrice-weekly dosing immediately post-haemodialysis for the full duration of their intensive phase. No patients had any evidence of neurotoxicity, all were prescribed oral eradication therapy, and none had relapsed infection, although one patient relocated to another region of Australia during their eradication phase and was lost to follow-up.

**Table 1.** Characteristics of patients with melioidosis receiving CIHD treated with ceftazidime, with pre-dialysis concentrations performed at Cairns Hospital, 2016–25

Age and sex	Weight (kg)^a^	Site of infection	Ceftazidime MIC (mg/L)^b^	Duration of IV treatment (weeks)	Haemodialysis regimen^c^	Residual urine output^d^	Ceftazidime dose^e^	Ceftazidime pre-dialysis concentration (mg/L)^f,g^	Ceftazidime toxicity	Outcome
62M	117	Bacteraemia, pulmonary	1	4	Thrice weekly	Minimal	2 g daily 1 g daily	88.7–126.1 56.7	No	Lost to follow-up
69F	69	Pulmonary, renal abscess	1	4	Thrice weekly	None	2 g daily 1 g daily	177.7–193.3 119.8–184.2	No	Cure
65M	79	Bacteraemia, prostate abscess	1	4	Thrice weekly	Minimal	2 g daily 1 g daily	128.7 57.2–62.0	No	Cure
80M	75	Bacteraemia, pulmonary	2	3	Thrice weekly	Minimal	2 g daily 2 g/2 g/3 g^h^	46.9–111.5 32.7–41.3	No	Cure
64F	73	Bacteraemia, pulmonary	1	4	Thrice weekly	Minimal	2 g daily	76.3	No	Cure
54M	148	Bacteraemia, pulmonary, urinary, myositis	1	4	Thrice weekly	Minimal	2 g daily	59.7–74.0	No	Cure
52M	68	Bacteraemia, pulmonary	1	3	Thrice weekly	None	2 g daily 2 g/2 g/3 g^i^	45.5–55.1 N/A	No	Cure
43F	82	Bacteraemia, pulmonary	1	3	Thrice weekly	1 L/day	2 g/2 g/3 g^j^	45.7	No	Cure
59F	105	Pulmonary, osteomyelitis	1	6	Twice weekly	1 L/day	2 g daily	57.8–63.5	No	Cure^k^

^a^Measured as dry weight.

^b^Defined by ETEST.

^c^All patients received CIHD on high-flux machines; regimen at time of ceftazidime pre-dialysis concentration monitoring.

^d^As estimated and reported by patient.

^e^Intermittent dosing; administered immediately post-haemodialysis.

^f^Range provided if more than one pre-dialysis concentration had been measured.

^g^Measured as free (unbound) concentration.

^h^2 g/2 g/3 g regimen given for 1 week.

^i^2 g/2 g/3 g regimen given for 1 week.

^j^2 g/2 g/3 g regimen given for 3 weeks.

^k^Currently completing oral eradication phase therapy.

## Discussion

This study suggests that in patients receiving CIHD, administering ceftazidime with a dosing regimen of 2 g/2 g/3 g thrice-weekly administered immediately post-haemodialysis achieves adequate serum pre-dialysis concentrations, can achieve good clinical outcomes and may be a potential alternative treatment approach for people with melioidosis once validated in a larger cohort.

Prolonged IV antibiotics are essential in the management of patients with melioidosis, and this is believed to at least partly explain the low rates of relapsed disease now seen in northern Australia.^14^ Patients receiving CIHD often have challenges with vascular access or need to preserve veins for future arteriovenous fistula formation, thereby making avoidance of recurrent peripheral IV access, including PICC insertion, desirable.^15^ Additionally, patients receiving CIHD are at increased risk of infections and have higher infection-related mortality, often due to organisms acquired during vascular access.^16^ While PICCs are associated with low rates of complications when managed by experienced healthcare workers, bloodstream infections, thrombosis and local complications can still occur.^17^ Administering ceftazidime immediately post-haemodialysis through existing vascular access is therefore welcome as it obviates the need for PICC insertion and prevents the risks and costs associated with this invasive procedure.

The incidence of melioidosis is increasing in our region, with almost 80% of cases occurring during the December–April wet season.^18^ This creates an expanded requirement for health system resources during this 5 month period, including hospital bed capacity and access to ICU support, sophisticated medical imaging and surgical services. Indeed, access to well-resourced hospital care has resulted in the case-fatality rate of melioidosis now declining to less than 10%.^19^ However, the extended duration of IV antibiotic therapy that is necessary to achieve these outcomes means that most patients choose to complete their course of IV therapy in their own home, utilizing our OPAT service. The OPAT service requires daily home visits by senior nursing staff, motor vehicles and oversight from pharmacists and infectious diseases physicians. The increased incidence and improving case-fatality rate of melioidosis has resulted in an increased demand for this necessarily finite service, which also must provide care to individuals with other diagnoses. Any alternative effective methods for treating people with melioidosis with IV antibiotics post-hospital discharge are therefore welcome. Patients receiving CIHD already attend hospital or a dedicated haemodialysis unit thrice weekly, and therefore administering post-haemodialysis ceftazidime reduces inconvenience for patients and allows the OPAT service to care for other patients.

Ceftazidime has a wide therapeutic range; however, neurotoxicity—manifested by confusion, myoclonic jerks and seizures—can occur. Thresholds for ceftazidime toxicity are incompletely defined, although a neurotoxicity threshold of 78 mg/L has been proposed.^20^ It is notable that half of the patients in this series who were initially administered 2 g daily had pre-dialysis concentrations that greatly exceeded this threshold, and over half underwent a dose or dosing frequency reduction. Despite pre-dialysis concentrations exceeding this proposed threshold in four of our patients, none experienced neurotoxicity. The pre-dialysis concentrations observed in this study were well above the 60%–70% *fT*_>MIC_ target, suggesting that lower doses of ceftazidime may be appropriate in some patients.

The retrospective nature of our study relied on documentation of ceftazidime pre-dialysis concentrations, and while these were cross-checked with timing of sample collection and commencement of haemodialysis, there may have been small delays between these times. The half-life of ceftazidime is prolonged in people with end-stage renal disease and some trough concentrations may have been measured before reaching steady state. However, early sampling in people receiving CIHD has utility as it can reassure clinicians that target attainment has been reached. Furthermore, the high concentrations observed in this study suggest that subtherapeutic dosing is extremely unlikely. Signs and symptoms of neurotoxicity may be subtle and not always reflected in clinical documentation. Nevertheless, these issues represent the challenges of therapeutic drug monitoring and antibiotic prescribing in the real-world setting of a busy referral hospital. Relapsed melioidosis in patients administered prolonged IV antibiotics is rare but can occur at a median of 9 months. There were no cases of relapsed disease in this series; however, four patients—all of whom remain well—are yet to reach this milestone.

The increasing availability of therapeutic drug monitoring and clinical breakpoints for *B. pseudomallei* suggest a more individualized approach is possible in people with renal impairment and melioidosis and, indeed, other infections. This will minimize potential toxicity while also ensuring good clinical outcomes. In people requiring prolonged IV antibiotics who are receiving CIHD, it may also allow avoidance of PICC insertion and their associated complications.
